# Propensity score to detect baseline imbalance in cluster randomized trials: the role of the c-statistic

**DOI:** 10.1186/s12874-015-0100-4

**Published:** 2016-01-22

**Authors:** Clémence Leyrat, Agnès Caille, Yohann Foucher, Bruno Giraudeau

**Affiliations:** INSERM U1153, Paris, France; INSERM CIC 1415, Tours, France; CHRU de Tours, Tours, France; Department of Medical Statistics, London School of Hygiene and Tropical Medicine, London, United Kingdom; Université François-Rabelais, PRES Centre-Val de Loire Université, Tours, France; SPHERE (EA 4275): Biostatistics, Clinical Research and Subjective Measures in Health Sciences, Université de Nantes, Nantes, France

**Keywords:** Cluster randomized trial, Confounding bias, Propensity score, *C-statistic*, Baseline imbalance

## Abstract

**Background:**

Despite randomization, baseline imbalance and confounding bias may occur in cluster randomized trials (CRTs). Covariate imbalance may jeopardize the validity of statistical inferences if they occur on prognostic factors. Thus, the diagnosis of a such imbalance is essential to adjust statistical analysis if required.

**Methods:**

We developed a tool based on the *c-statistic* of the propensity score (PS) model to detect global baseline covariate imbalance in CRTs and assess the risk of confounding bias. We performed a simulation study to assess the performance of the proposed tool and applied this method to analyze the data from 2 published CRTs.

**Results:**

The proposed method had good performance for large sample sizes (n =500 per arm) and when the number of unbalanced covariates was not too small as compared with the total number of baseline covariates (≥40 *%* of unbalanced covariates). We also provide a strategy for pre selection of the covariates needed to be included in the PS model to enhance imbalance detection.

**Conclusion:**

The proposed tool could be useful in deciding whether covariate adjustment is required before performing statistical analyses of CRTs.

**Electronic supplementary material:**

The online version of this article (doi:10.1186/s12874-015-0100-4) contains supplementary material, which is available to authorized users.

## Background

In cluster randomized trials (CRTs), the units of randomization are not individuals but rather the social units to which the individuals belong [1]. This may challenge the balance between groups in terms of baseline covariates. Indeed, clusters are sometimes randomized before the identification and recruitment of participants, which may jeopardize allocation concealment [2–5]. In their review, Puffer et al. [6] showed that 39 % of the selected CRTs were at risk of confounding bias on individual characteristics. That was also supported by the work of Brierley et al. [7], who found a risk of bias in 40 % of CRTs that did not use prior identification of participants. In addition, the risk of chance imbalances increases when the number of randomized clusters decreases, which is frequent [8, 9].

Some allocation techniques have been proposed to achieve a better baseline balance in CRTs, but they are not always feasible to implement in practice [10]. If imbalance occurs on one or more prognostic factors, the intervention effect estimate may be biased and could compromise the validity of statistical inferences. Identifying baseline imbalance in CRTs is therefore of importance to implement suitable statistical analyses.

In individually randomized trials, statistical testing is not recommended to assess group comparability because if randomization is properly applied, all observed imbalances will be due to chance [11, 12]. When reporting the results of a randomized trial, the CONSORT statement advises displaying baseline characteristics in a table to gauge group comparability [13]. The same recommendation is given in the CONSORT extension for CRTs, both for individual-level and cluster-level covariates [14]. Fayers and King [15] stated that significance tests *“are usually only worth doing if potential violation of the randomisation is suspected”*. In some CRTs, allocation concealment is impossible (i.e., when, for instance, participants are recruited after the randomization of clusters, and because no blinding is possible), and therefore, in this case, tests may be worthwhile. Nevertheless, Wright et al. [16] showed that about 44.7 % of papers reporting the results of a CRT did not provide a statistical test for covariate balance, and 20 % did not even display a table reporting covariates between groups.

The problem of baseline imbalance observed in some CRTs is close to the imbalance that can occur in observational studies [17]. For these latter studies, several methods exist to assess group comparability at baseline. The methods can be divided into two groups: those that assess covariate balance one by one, and those that allow a global assessment of the balance on several baseline covariates [18]. Significance testing (based on *t* test or *χ*^2^ test, for example), standardized difference [19], overlapping coefficient [18] and Kolmogorov-Smirnov [20] or Lévy [21] distances are in the first group of methods. Belister [22] found that the standardized difference (see Table 1) had the highest correlation with the bias of the intervention effect estimate. Standardized differences also perform well with small sample sizes [23], so it may have the best performance in detecting baseline imbalance when covariates are considered one by one. Nevertheless, this method does not provide a global overview of the overlap of covariates between groups. Global assessment of imbalance on several covariates simultaneously is of interest in that it allows for capturing the correlations between covariates. For example, let us consider two quantitative prognostic factors, for which the impact on the outcome is on the same direction: high values for these covariates lead to a higher risk of an event. Because each of these prognostic factors is slightly unbalanced, a univariate test may not detect any imbalance. However, the impact of both imbalances together may cause an important bias in the intervention effect estimate. Consequently, a global approach is more appropriate in the context of CRTs to handle complex relationships underlying a potential confounding bias.

**Table 1:** Standardized differences

Baseline groups comparability for each covariate can be assessed with the standardized difference [19]. For a continuous covariates *X*, the standardized difference SD is:
$\label {diff_stand} \text {SD}=\frac {100 \times \left |\bar {x}_{1}-\bar {x}_{0}\right |}{\sqrt {\frac {{s_{0}^{2}}+{s_{1}^{2}}}{2}}}, \qquad \qquad \qquad \qquad \qquad \qquad \qquad \qquad (1) $
where $\bar {x}_{0}$ and $\bar {x}_{1}$ are *X* means in control and intervention arm, respectively, and ${s^{2}_{0}}$ and ${s^{2}_{1}}$ the corresponding variance estimates. For a binary covariate, the SD is expressed as follows:
$\label {diff_stand_bin} \text {SD}=\frac {100 \times \left |\hat {P}_{1}-\hat {P}_{0}\right |}{\sqrt {\frac {\hat {P}_{0} (1-\hat {P}_{0})+\hat {P}_{1}(1-\hat {P}_{1})}{2}}},\qquad \qquad \qquad \qquad \qquad \qquad \qquad \,\,\,(2) $
where $\hat {P}_{0}$ and $\hat {P}_{1}$ are the observed rates for the covariate in control and intervention arm, respectively. The strength of SD as compared to statistical tests is that this measure does not depend on the sample size nor on the measurement scale [50]. Usually, covariates with a SD exceeding 10 % are considered to be unbalanced [41]. However, for binary covariates, a SD of 10 % can sometimes be negligible [51].

Global metrics include the Mahalanobis distance [24], the post-matching *c-statistic* of the propensity score (PS) model [18] and *L*^1^ measure [25]. Franklin et al. [18] found that the *c-statistic* of the PS model led to the better prediction of bias for binary, count or continuous outcome, provided the sample size is large enough. This statistic represents the extent to which covariates can predict intervention allocation. The *c-statistic* of the PS model has been used to help in the selection of variables to include in the PS model (even if this method is not recommended [26]) but to our knowledge has not been used as a tool to detect baseline imbalance.

In this context, we developed a decision rule based on the *c-statistic* of the PS model and its expected probability distribution to assess baseline imbalance. This method can be viewed as a global statistical testing at a 5 % significant level. The basic idea is to use the distribution of the *c-statistic* in accordance with the characteristics of the CRT (size, number of covariates) to choose the cut-off for the detection of imbalance, rather than using a unique threshold value. It is important to note that the PS model fitted in order to detect imbalance is different from the model fitted for the statistical analysis of the trial. In both situation, the outcome of the PS model is the treatment allocation, but, in the former situation, all covariates associated with treatment allocation have to be included in the PS model, whereas in the latter, covariates both linked to treatment allocation and the outcome need to be accounted for. Indeed, when analyzing the trial, selecting confounding only for a propensity score analysis is desirable [27, 28] while such a restriction does not hold for our aim which is to detect any baseline imbalance, to obtain a qualitative assessment of the risk of bias in a given CRT. This paper is organized as follows. We first describe two CRTs motivating examples at risk of confounding bias because clusters were randomized before the patients were enrolled. We then give the theoretical background for the PS approach and the *c-statistic*, followed by the objectives of the present paper and the principle of our method. Then, we give the design and the results of a simulation study to assess the performance of our method based on the distribution of the *c-statistic* to detect baseline imbalance in CRTs. The implication in terms of risk of confounding bias and need for covariates adjustment are then discussed, along with an application of our method with the two motivating examples.

### Motivating examples

#### Example 1: Management of osteoarthritis with a patient-administered assessment tool

The first motivating example was a published CRT using a 2×2 factorial design that aimed to assess the impact of an unsupervised home-based exercise programme, the use of standardized evaluation tools, their combination, or usual care on symptoms (pain, global assessment of disease and physical functioning) in patients with knee and hip osteoarthritis (OA) [29]. A total of 867 rheumatologists were randomized and each had to enrol four patients (three with knee OA and one with hip OA). Thus, rheumatologists were not blinded to intervention allocation. For simplicity, we focus on only one intervention: the use of standardized evaluation tools. In all, 1462 patients received the standardised evaluation tools and 1 495 patients received usual care. Twelve covariates were collected at baseline (Table 2). Standardized differences are displayed to assess the balance between arms. Univariate statistical testing showed an imbalance in age, pain, disability (measured by the the Western Ontario McMaster University Osteoarthritis Index [WOMAC] physical function subscale) and global assessment of disease at a 5 % significance level. These imbalances correspond to a standardized difference of 7.81 *%* for age but greater than 10 *%* for the other variables. Moreover, these variables were known to be strongly associated with the potential outcome of the subjects. Because these variables were associated with whether patients were enrolled into the trial in a given group, they constituted possible confounders. In addition, pain, WOMAC score and global assessment of disease were correlated with each other, with Pearson correlation coefficients in the range $\left [0.38-0.49\right ]$.

**Table 2:** Patient baseline characteristics per group in the study on management of osteoarthritis with a standardized evaluation tool (first motivating example)

Characteristics	Control	Standardized tool	*p*	SDiff (%)
	*n*_0_=1495	*n*_1_=1462		
	*mean (standard deviation)*	*mean (standard deviation)*		
Duration of symptoms (months)	68.0 (69.8)	70.9 (75.0)	0.2737	4.03
Age (years)	67.2 (9.7)	66.4 (10.0)	**0.0344**	7.81
BMI (kg.m ^−2^)	27.8 (4.9)	27.7 (4.7)	0.3824	3.14
Patient global assessment (0-100)	61.1 (18.2)	56.7 (17.4)	**< 0.0001**	24.44
Pain evaluation VAS (0-100)	59.4 (16.0)	55.3 (15.1)	**< 0.0001**	26.32
WOMAC function score (0-100)	45.5 (16.3)	43.8 (16.0)	**0.0050**	10.32
	*n (%)*	*n (%)*		
Osteoarthritis in other joints	1366 (91.4)	1311 (89.7)	0.1297	5.81
Prior treatment				
IA treatment	434 (29.0)	432 (29.6)	0.7877	1.14
NSAIDs	955 (63.9)	958 (65.5)	0.3689	3.45
SYSADOA	617 (41.3)	605 (41.4)	0.9810	0.22
Male	424 (28.4)	459 (31.4)	0.0782	6.65
Kellgren and Lawrence grade			0.2594	
III	724 (48.4)	677 (46.3)		4.25
IV	516 (34.5)	547 (37.4)		6.02

BMI: Body Mass Index; VAS: Visual Analogue Scale; WOMAC: Western Ontario and McMaster Universities Arthritis Index; IA: intra-articular; NSAID: non-steroidal anti-inflammatory drug; SYSADOA: systematic slow acting drug for osteoarthritis. SDiff: standardized difference; *p*: *p*-value for univariate tests (adjusted *t* test for quantitative variables, adjusted chi-square test for qualitative variables to take the clustering into account). Bold values are significant tests at a 5 % significance level

#### Example 2: Standardized consultation for patients with osteoarthritis of the knee

The second example was a CRT which evaluated the impact of standardized consultations on patients with OA of the knee versus usual care [30]. It was an open pragmatic CRT in which 198 rheumatologists were randomized, each of whom had to include two consecutive patients who met the inclusion criteria. In total, 154 patients were allocated to standardized consultation and 182 to usual care. Overall, 26 covariates were measured at baseline (Table 3). Statistical testing revealed a significant imbalance in body mass index (BMI), delay in years since the beginning of pain, age at the beginning of pain and the use of non-drug treatments. Moreover, some other variables (weight, pain, Medical Outcomes Study Short Form 12 [SF-12] mental, and eight concommitant treatments) had a standardized difference greater than the usual threshold of 10 %.

**Table 3:** Patient baseline characteristics per group in the study on standardized consultation for patients with osteoarthritis of the knee (second motivating example)

Characteristics	Control	Standardized consultation	*p*	SDiff (%)
	***n***_**0**_ **=146**	*n*_1_=181		
	*mean (standard deviation)*	*mean (standard deviation)*		
Age (years)	64.5 (8.4)	63.9 (8.1)	0.4720	8.02
Weight (kg)	81.4 (13.6)	84.1 (12.9)	0.0665	20.60
BMI (kg.m ^−2^)	30.2 (3.8)	31.2 (3.5)	**0.0143**	27.63
PEL (0-5)	2.2 (0.9)	2.2 (0.8)	0.9594	0.00
Delay since beginning of pain (years)	5.5 (5.9)	7.4 (7.5)	**0.0152**	27.57
Age at beginning of pain (years)	59.1 (10.4)	56.5 (10.5)	**0.0300**	24.28
Pain (0-10)	5.6 (1.3)	5.5 (1.2)	0.3646	10.38
WOMAC function score (0-100)	29.9 (12.2)	30.3 (11.7)	0.7377	3.76
SF-12 physical subscale	34.8 (6.7)	35.4 (6.7)	0.4385	8.70
SF-12 mental subscale	41.4 (9.4)	43.3 (10.1)	0.0827	19.31
Global assessment of disease status (0-10)	5.6 (1.5)	5.6 (1.5)	0.9133	1.31
	*n (%)*	*n (%)*		
Male	49 (27.1)	34 (23.3)	0.5132	8.73
Prior treatments				
Analgesics	130 (71.8)	96 (65.8)	0.2889	13.13
NSAIDs	95 (52.5)	90 (61.6)	0.1215	18.58
Current use of NSAIDS	160 (88.4)	126 (86.3)	0.6883	6.31
SYSADOA	74 (40.9)	68 (46.6)	0.3576	11.49
Current use of SYSADOA	179 (98.9)	145 (99.3)	1.0000	4.46
IA treatment	31 (17.1)	29 (19.9)	0.6229	7.05
Non-drug treatment	110 (60.8)	71 (48.6)	**0.0372**	24.58
Diet	49 (27.1)	31 (21.2)	0.2750	13.67
Dietetician	12 (6.6)	7 (4.8)	0.6401	7.91
Physical exercice	44 (24.3)	27 (18.5)	0.2571	14.22
Physiotherapy	30 (16.6)	17 (11.6)	0.2692	14.20
Knee orthosis	21 (11.6)	11 (7.5)	0.2967	13.86
Insoles	24 (13.3)	11 (7.5)	0.1376	18.84
Walking sticks	13 (7.2)	10 (6.8)	1.0000	1.30

BMI: Body Mass Index; PEL: Baecke’s physical exercice level scale; WOMAC: Western Ontario and McMaster Universities Arthritis Index; SF-12: 12-items Short Form; IA: intra-articular; NSAID: non-steroidal anti-inflammatory drug; SYSADOA: systematic slow acting drug for osteoarthritis. SDiff: standardized difference; *p*: *p*-value for univariate tests (adjusted *t* test for quantitative variables, adjusted chi-square test for qualitative variables to take the clustering into account). Bold values are significant tests at a 5 % significance level

### Theoretical background

#### Propensity score theory

The PS theory was initially developed by Rosenbaum and Rubin [31] to overcome the problem of confounding bias in observational studies. The individual PS refers to the individual probability, for a subject *l* involved in a study, of receiving the intervention of interest (*T*_*l*_=1) rather than the control intervention (*T*_*l*_=0), conditionally on the subject’s characteristics at baseline ***x***_***l***_=(*x*_(1)*l*_,…,*x*_(*r*)*l*_). The PS is frequently denoted by *e*(***x***_***l***_) and is defined as *e*(***x***_***l***_)=*P*(*T*_*l*_=1|***x***_***l***_). The true PS is unknown in practice, but it can be estimated by logistic regression, modeling the probability of receiving the intervention of interest given *r* observed covariates as follows:

(3) $$ e(\boldsymbol{x_{l}})=\left\{1+\exp{\left(-\alpha_{0}-\sum_{p=1}^{r}{\alpha_{p}x_{(p)l}}\right)}\right\}^{-1},   $$

where *α*_0_ is the intercept and *α*_*p*_(*p*=1,…,*r*) are the regression coefficients.

In CRTs, the PS has been studied for the estimation of the intervention effect [27, 28], or to improve randomization [32] but not for detection of imbalance between groups.

#### The *c-statistic*

The *c-statistic* (concordance statistic) measures the discriminatory capacity of a predictor [33]. It also corresponds to the area under the receiver operating characteristic (ROC) curve, which displays sensitivity as a function of 1-specificity for all the possible thresholds of the predictor [34]. If we consider an intervention allocation (intervention *vs.* control), the *c-statistic* is the probability that a subject receiving the intervention has a higher value for the predictor than a subject in the control group [35]. It can be estimated as follows: 
(4)

where *i*=1,…,*n*_0_ is the participant index in the untreated group, *j*=1,…,*n*_1_ is the participant index in the treated group and *𝟙* is a dummy variable equals to 1 if *p*_*i*_<*p*_*j*_, 0 otherwise. The *c-statistic* takes its values in the range [0.5;1.0], where 0.5 corresponds to a classification that does not outperform chance and 1.0 corresponds to perfect classification. In our situation, the groups are the treatment arms and the predictor is the prediction obtained from the PS model. The *c-statistic* is often computed with the predictions obtained from a logistic model.

#### Propensity scores and the *c-statistic*

In the absence of baseline imbalance, the PS has a normal distribution of mean 0.5 in each group of the study, and thus the baseline variables are independant from the intervention allocation. In other words, the *c-statistic* of the PS model (3) is close to 0.5. By contrast, if at least one covariate is associated with intervention allocation, the *c-statistic* will be larger than 0.5. To our knowledge, the *c-statistic* of the PS model has not been used as a tool to detect baseline imbalance.

### Objectives and principles

We developed a method, based on the *c-statistic* of the PS model, to detect baseline imbalance between groups in CRTs. In practice, this method is a tool to appreciate the risk of confounding bias and to identify the situations in which suitable statistical methods to take imbalance into account must be implemented. Our method relies on three steps: **(i)** The *c-statistic* is estimated from the data of the CRT for which one wants to assess the baseline balance **(ii)** The 95^th^ percentile of the *c-statistic* distribution under the hypothesis of no systematic baseline imbalance is determined from simulation with the same number of covariates and sample size in the CRT **(iii)** The statistical decision rule is expressed as follows: if the *c-statistic* estimated in step (i) is above the threshold value obtained in step (ii), then a baseline imbalance is suspected.

Because of the use of the 95^th^ percentile of the *c-statistic* distribution as a threshold, our method is similar to a global statistical test for baseline imbalance at a 5 % significance level. It is important to note that our method focuses only on individual-level characteristics; indeed, in CRTs, clusters are the unit of randomization and thus any observed imbalance in cluster-level covariates will be due to sampling fluctuations. Applying this method to cluster-level covariates would be similar to baseline tests for individually randomized trials, which is not recommended in practice. Conversely, because participants are not the randomization units in CRTs, confounding bias may affect some trials, leading to systematic imbalances in individual-level variables [17]. Because threshold values are different for each combination of sample size and number of covariates involved (illustrative results are given in Additional file 1), our approach is more flexible than using a unique threshold for the *c-statistic*. Indeed, our methods uses the empirical distribution of the *c-statistic* of the PS model considering the characteristics of the CRT of interest.

The objectives of the present paper are to assess the performance of this method with a simulation study and to interpret the diagnosis of baseline imbalance in terms of risk of confounding bias and need for covariate adjustment.

## Methods

### Design of the simulation study

We performed a simulation study to assess the performance of the proposed method to detect baseline imbalance. The determination of thresholds values for our methods (step (ii) in the principle of our method) is described in Additional file 1: Appendix A.

### Data generation

We generated datasets corresponding to CRTs without systematic imbalance and estimated the *c-statistic* of the PS model for each dataset. The data were generated as follows:

- **Cluster size**: Let us consider a two-parallel-arm CRT, in which 2*k* clusters of mean size *m* are randomized. We generated cluster sizes, as proposed by Turner et al. [36], from a Poisson distribution with parameter *m*: $m_{\textit {ij}}\sim \mathcal {P}(m)$, (*i*=0,1 the intervention index and *j*=1,…,*k* the cluster index).
- **Covariates**: Let ***X***=(*X*_1_,…,*X*_*r*_) be a vector of *r* randomly generated covariates, among which *r*_*c*_ are continuous covariates and *r*_*b*_ are binary (*r*_*c*_+*r*_*b*_=*r*). To generate ***X***, a vector ***X***_***0***_ was first drawn in a multivariate normal distribution $\mathcal {N}_{r}\sim (0,\boldsymbol {\Sigma _{r\times r}})$, without loss of generalizability.
  At this stage, we have a matrix ***X***_***0***_ of *r* continuous balanced covariates measured at baseline. However, this situation does not differ from an individually randomized trial. To fit to the situation of a real CRT, we induced an intraclass correlation for the covariates meaning that subjects belonging to the same cluster had more similar individual characteristics. We randomly drew a cluster effect *γ*_*pj*_, (*j*=1,…,*k*) for each cluster *j* and each covariate *p* (*p*=1,…,*r*) in a distribution $\mathcal {N}(0,0.15)$, with the constraint $\sum _{j=1}^{k}{\gamma _{\textit {pj}}}=0$ in each arm. The variance parameter for the cluster effect of 0.15 was chosen to obtain intraclass correlation coefficient (ICC) values for the covariates in the range [0.01;0.05]. These values are based on those observed for baseline characteristics in the study of Kul et al. [37]. Then, for each subject in cluster *j* and each covariate, a random error was drawn from a distribution $\mathcal {N}(\gamma _{\textit {pj}},1)$ and this error term was added to ***X***_***0***_, the initial value of the covariate.
  Among the *r* generated covariates, we induced an imbalance on *s* of them. These covariates were correlated with each other, because such correlations are often observed in clinical trials [38]. Moreover, for each of the *s* unbalanced covariates, the standardized difference (reflecting the imbalance ‘size’) depended on the degree of correlation between covariates: two highly correlated covariates had similar standardized differences. To induce the correlations between the standardized differences, a vector ***X***_***0***_=(*X*_1_,…,*X*_*r*_) of *r* covariates was first randomly drawn from a multivariate normal distribution $\mathcal {N}_{r}\sim (0,\boldsymbol {\Sigma _{r\times r}})$ with the following covariance matrix:
  $${} \boldsymbol{\Sigma_{r\times r}= \left(\begin{array}{ccccccc} 1 & \sigma_{1,2} & \cdots & \sigma_{1,s} & \sigma_{1,s+1} & \cdots & \sigma_{1,r}\\ \sigma_{2,1} & 1 & \cdots & \sigma_{2,s} & \sigma_{2,s+1} & \cdots & \sigma_{2,r}\\ \vdots & \vdots & \ddots &\vdots &\vdots &\cdots &\vdots\\ \sigma_{s,1} & \sigma_{s,2} & \cdots & 1 & \sigma_{s,s+1} & \cdots & \sigma_{s,r}\\ \sigma_{s+1,1} & \sigma_{s+1,2} & \cdots & \sigma_{s+1,s} & 1 & \cdots & \sigma_{s+1,r} \\ \vdots & \vdots & \cdots &\vdots & \vdots &\ddots &\vdots\\ \sigma_{r,1} & \sigma_{r,2} & \cdots &\sigma_{r,s} &\sigma_{r,s+1}& \cdots & 1\\ \end{array}\right)}.$$
  *σ*_*f*,*g*_, *f*, *g*=1,..,*q*, *f*≠*g* represents the covariance and the correlation between covariates *X*_*f*_ and *X*_*g*_ in that the covariates followed standard normal distributions. The covariance matrix ***Σ***_***r×r***_ was a positive definite matrix randomly generated with the R function *genPositiveDefMat* from the *clusterGeneration* package. For convenience, we considered the absolute values of the covariance matrix.
  Second, the sub-matrix ***Σ***_***s×s***_ of ***Σ***_***r×r***_ was used to draw the standardized differences for unbalanced covariates from a multivariate normal distribution. Let *Δ* and *s*_*Δ*_ be respectively the mean standardized differences of unbalanced covariates and its standard deviations. Thus, the *s* unbalanced covariates followed a distribution $\mathcal {N}(\Delta,s_{\Delta }^{2})$. As *σ*_*f*,*g*_=*r*_*f*,*g*_×*σ*_*j*_*σ*_*k*_, the covariance matrix $\boldsymbol {\Sigma _{s\times s}^{\Delta }}$ used to generate the standardized differences was: $ \boldsymbol {\Sigma _{s\times s}^{\Delta }}= s_{\Delta }^{2} \boldsymbol {\Sigma _{s\times s}}. $ Thus, standardized differences ***Δ***=(*Δ*_1_,…,*Δ*_*s*_) were drawn from a multivariate normal distribution with mean ***Δ****𝟙*_*s*_ with $\boldsymbol {\Sigma _{s\times s}^{\Delta }}$ for the covariance matrix. Then, for an unbalanced covariate *f* (*f*=1,…,*s*) and a subject *l*, the covariate’s value was ***X***_***fl***_+***Δ***×*T*_*l*_, where ***X***_***fl***_ corresponded to the *f*^*th*^ covariate’s value for subject *l* when generating *X*_0_ and *T*_*l*_ being the intervention indicator for subject *l*, as previously defined.
  Finally, *r*_*b*_ covariates from ***X***_***0***_ were dichotomized by covariate-specific threshold values *t*_*p*_(*p*=1,…,*r*_*b*_). Thresholds *t*_*p*_ were *a priori* fixed to obtain the desired prevalences *P*_*p*_ of these characteristics, drawn in a uniform distribution in the range [ 0.2;0.8]. From *P*_*p*_, the threshold was: *t*_*p*_=*Φ*^−1^(1−*P*_*p*_), where *Φ* is the cumulative density function (CDF) of a standard normal distribution. Doing so, the standardized difference for binary covariates could be calculated from the formula in Table 1 with $\hat {P}_{1_{b}}=\Phi (\Phi ^{-1} (1-\hat {P}_{0_{b}})-\Delta /100)$, where $\hat {P}_{0b}$ and $\hat {P}_{1b}$ are the observed proportions in the control and the intervention arms, respectively.

### Propensity score estimation

The PS was estimated with a logistic model adjusted on the set of generated covariates. A cluster-specific random effect cannot be taken into account in this model because clusters are nested in the intervention arm (subjects from the same cluster received the same intervention). Even if this limitation can have an impact when PS is used to estimate the intervention effect [28], the impact on the performance for imbalance detection is negligible because clusters are the unit of randomization. Thus, considering clusters as a fixed effect, cluster effect would be balanced between groups.

### Covariate pre-selection

Within the simulation, we also proposed two criteria to select only some covariates among the *r* generated covariates, in order to assess the efficiency of a more parsimonious model to detect imbalance, because numerous studies showed the importance of covariate selection for PS model to avoid over-fitting problems [39, 40]. Moreover, the presence of a large number of balanced covariates in the PS model can attenuate the importance of a potential global imbalance. A covariate was included in the PS model if it satisfied at least one of these two criteria:

- its standardized difference was ≥5 *%*
- its standardized difference was <5 *%* but its correlation with at least one covariate with a standard difference ≥ 5 % was greater or equal to 0.2 in absolute value.

These criteria allowed for selecting covariates with more flexibility than with univariate testing. In practice, a covariate is supposed to be unbalanced when its standardized difference ≥ 10 % [41], whereas our method was less stringent for the number of covariates kept. Moreover, a balanced covariate highly correlated with an unbalanced one may have an impact on the *c-statistic*. This strategy allowed to assess if baseline imbalance must be diagnosed from all available baseline covariates.

## Threshold value

For each studied scenario, the corresponding threshold value to conclude to baseline imbalance was obtained from simulations with the same simulation parameters but under the hypothesis of no systematic imbalance (i.e. the *r* generated covariates are balanced). The impact of sample size, number of clusters, number of covariates and trial design (CRT or individually randomized trial) on the *c-statistic* of the PS model without systematic imbalance was studied beforehand and results are presented in Additional file 1: Appendix A.

## Results assessment

The results were assessed in terms of the following:

- proportion *π* of simulated datasets in which the estimated *c-statistic* was greater or equal to the threshold value defined as the 95th percentile of the *c-statistic* distribution in absence of systematic baseline imbalance, *i.e.,* the proportion of situations in which baseline imbalance was detected, according to our proposed rule,
- for each unbalanced covariate, the proportion of significant univariate tests at a 5 % significance level. These tests were adjusted *t* test and adjusted chi-square test, described in [1] to take the clustering into account.

## Studied scenarios

First, we studied 144 scenarios corresponding to the different combination of the following parameters:

- **the sample size per arm**: *n*=(100,500). In CRTs, the median number of subjects per arm is 329 (interquartile range [143–866]) [42]. Thus, the chosen values correspond to the situation of a small and average size CRT.
- **the number of clusters per arm**: *k*=(5,10,50),
- **the number of covariates**: *r*=(4,10,20) for *n*=100 and *r*=(10,20,50) for *n*=500, corresponding to ratios $\frac {n}{r}=(25,10,5)$ for *n*=100 and $\frac {n}{r}=(50, 25, 10)$ for *n*=500. We considered $r_{c}=r_{b}=\frac {r}{2}$.
- **the number of unbalanced covariates**: *s* was defined such that the percentage of unbalanced covariates among all covariates was 20 % or 40 % (except for the case *k*=5, *m*=20 in which 25 % and 50 % of covariates were unbalanced). Thus, *s*=(2,4) for *r*=10, *s*=(4,8) for *r*=20 and *s*=(10,20) for *r*=50. Among unbalanced covariates, $\frac {s}{2}$ were binary and $\frac {s}{2}$ were continuous.
- **the standardized difference for unbalanced covariates**: *Δ*(*s*_*Δ*_)=10 *%*(5 *%*) or 20 *%*(10 *%*).

Second, we studied the performance of our method after covariate selection according to the rule expressed in Covariate pre-selection section of the main paper. We focused on scenarios in which the total number of covariates was ≥ 20 and the standardized difference for unbalanced covariates was moderate (10 %), corresponding to 36 different scenarios.

In both situations, we performed 5000 simulations.

## Results

### Results without covariate pre-selection

**Fig. 1:**
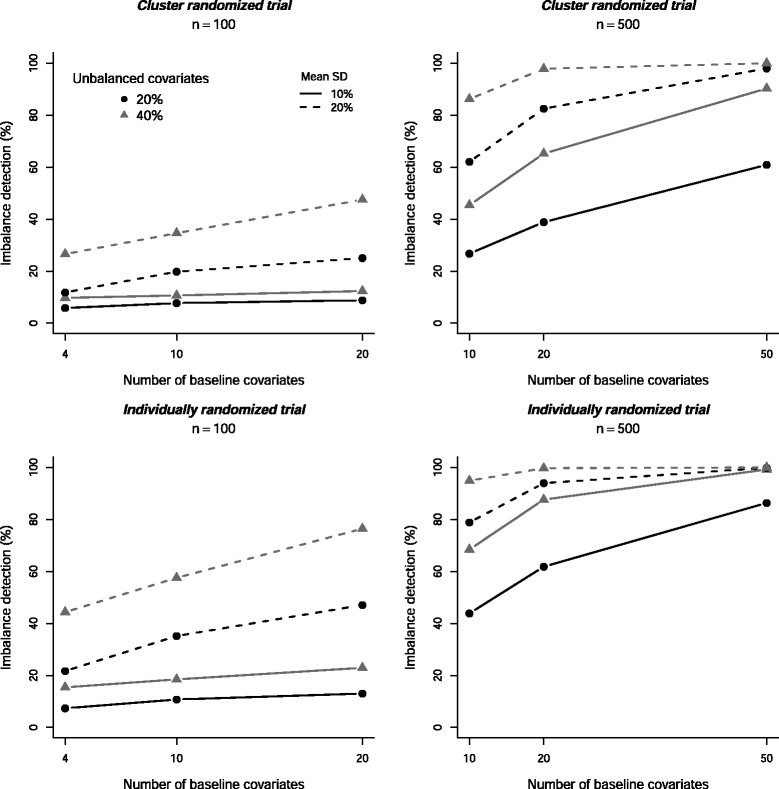
Percentage of imbalance detection *π* as a function of the number of baseline covariates *r*, the sample size per arm *n*, the standardized difference (SD) for unbalanced covariates and the percentage of unbalanced covariates 100×*s*/*r*. Results were pooled over the number of clusters per arm *k*. Five thousand simulations were performed per scenario

The results are displayed in (Fig. 1). As expected, the imbalance detection rate *π* (i.e. the proportion of situations in which our method allowed to detect imbalance) was higher when the standardized differences for the unbalanced covariates was high (20 %) than for a moderate imbalance (10 %). Second, imbalance was detected more often when the proportion of unbalanced total baseline covariates was higher 40 or 50 % (for *k*=5 and *m*=20) than when this proportion was 20 or 25 % (for *k*=5 and *m*=20). This result suggested that when there were too many balanced covariates, the information on unbalanced covariates was attenuated.

Moreover, the percentage imbalance detection was higher with sample size *n*=500 than with *n*=100. However, this latter situation corresponded to a small sample size (lower than the first quartile of the sample size per arm in a review of CRTs). This percentage increased also with the number of covariates. When the percentage of unbalanced covariates remained constant, the performance was better with increased number of total covariates (and thus the number of unbalanced ones), which suggests that the method allowed for capturing a global imbalance rather than imbalance on isolated covariates.

### Results with covariate pre-selection

For a set of 20 baseline covariates, the average number of covariates retained after the pre-selection was 13.5 with 20 % unbalanced covariates and 14.1 with 40 % unbalanced covariates. For a set of 50 baseline covariates, the average number of covariates retained was 27.1 and 29.7 with 20 and 40 % of imbalance, respectively. So this pre-selection mechanism allowed for retaining a large set of covariates, which was the basic idea for our method.

**Fig. 2:**
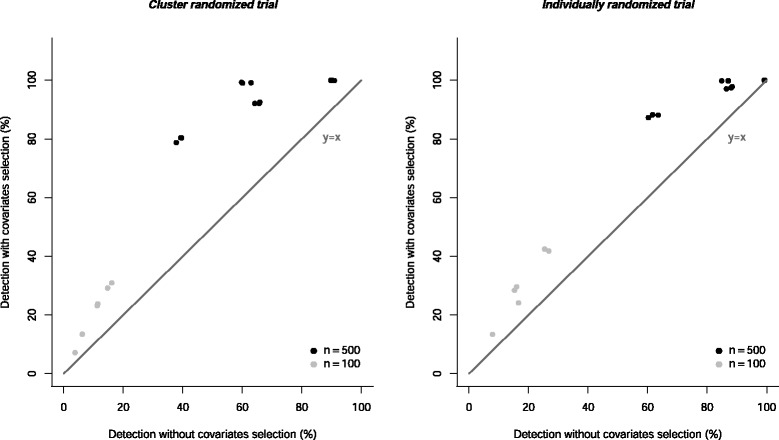
Percentage of imbalance detection *π* ^′^ after covariate pre-selection as a function of the initial percentage of imbalance detection *π*. Each point corresponds to a different number of covariates. The gray line is the first bisector. Five thousand simulations were performed per scenario

Moreover, this pre-selecting strategy for the covariates allowed for a systematic improvement in the percentage of bias detection for each study scenario, as displayed in Fig. 2. The relative improvement (defined as the ratio of the difference in percentage of imbalance detection with and without selection) varied from 0.7 % for a scenario in which the initial percentage of imbalance detection equaled 99.4 % before pre-selection, to 116.4 % for the scenario showing the worst performance without covariate pre-selection. However, this strategy was mainly helpful for scenarios in which the initial performance was moderate (about 50 %). Even after an average improvement >100 *%* for a small sample size (*n*=100), the performance remained <50 *%*. Indeed, in these situations the risk of chance imbalance due to sampling fluctuations is high (balance is achieved according the law of large numbers). Thus, threshold values for these trials are large even with no systematic imbalance and consequently, the detection rate is small. However, covariate selection increases detection rate in every scenario, so these results confirmed the need for a parsimonious PS model (i.e. including only a subset of covariates) that could be obtained with our simple and automatic proposed strategy.

### From global imbalance to confounding bias

Once the imbalance is detected, further assessment could be conducted to assess any risk of confounding bias, that is, if at least one of the covariates included in the PS model is also associated with the outcome. Such a variable, known as a confounding factor, is both associated with the intervention allocation and the outcome and may lead to a mis-estimation of the intervention effect [43]. Statistical measures of association can be used to identify them, as well as the literature to identify known confounders for a given outcome. When confounding bias is suspected, adjustment is required, whereas if the imbalance results from chance, adjustment would only improve the precision of the estimate, at least in linear models [44]. Among adjustment methods available for CRTs, multivariable regression [45] or PS-based methods [46, 47] are commonly used. However, the best predictive PS model is not the best model to correct imbalance [40]. As compared with a model for imbalance detection which can involve a large amount of covariates, a good PS model would include only confounding factors [39]; covariates which are related only to the intervention would increase standard errors without reducing bias [48]. A simulation study showed that discrimination criteria, such as the *c-statistic* or adequation tests such as Hosmer and Lemeshow cannot detect the omission of a confounding factor [26]. Consequently, the model built to detect imbalance is not the most proper for the statistical analysis.

**Fig. 3:**
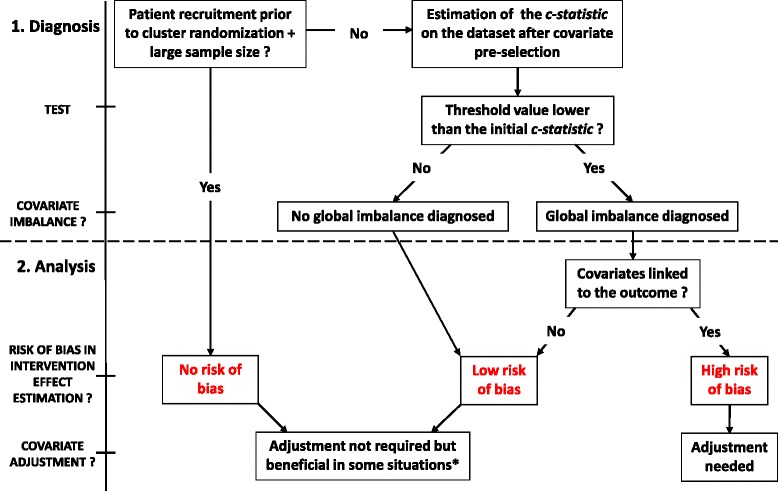
Steps for bias detection and guidance for covariates adjustment. Our diagnosis tool corresponds to the top part of the graph (part 1), whereas the bottom part (part 2) is a qualitative approach to help to perform a covariate adjustment. Part 2 has to be thought in accordance to clinical knowledge about potential confounders. *Adjustment on predictors can increase precision in linear model and generally increases power in case of chance imbalance

Figure 3 displays the different steps that help identifying the need of covariate adjustment. If patients are identified before cluster randomization and if the sample size is large enough, there is no risk of global imbalance or confounding bias and adjustment is not required. If cluster randomization occurs after patients recruitment but the sample size is small, there is a risk of chance imbalance. If cluster randomization occurs beforehand, there is a risk of systematic bias. In the last two situations, our tool can detect a global imbalance. If a such imbalance is detected, the assessment of the association between covariates and the outcome is needed to identify confounding bias, i.e. the presence of covariates both linked to the intervention and the outcome. When confounders are detected, covariate adjustment is needed to obtain an unbiased estimate of intervention effect. Otherwise, covariate adjustment will have no impact on the estimate but can increase precision for linear models.

### Results from the two motivating examples

For the two following examples, threshold values to detect baseline imbalance were obtained under a hypothesis of no systematic imbalance, with the same number of covariates (and the same proportion of continuous and binary covariates) and the same sample size as in the original CRT. For covariate generation, we used the observed mean (or rate) and standard deviations of covariates in the control arm and the correlation matrix from each CRT.

### Example 1: Management of OA with a patient-administered assessment tool

The PS was estimated with a logistic model adjusted on the 12 covariates displayed in Table 2. The PS distributions by arm are displayed in Additional file 1: Appendix C Figure 2a. The estimated *c-statistic* from this model was 0.598. The threshold value under the hypothesis of no systematic baseline imbalance was 0.549, below the estimated *c-statistic* for the dataset. We also applied our method using the pre-selection methods for the covariates: seven covariates among the 12 measured at baseline were retained. The estimated *c-statistic* was 0.595, and the corresponding threshold value was 0.541. Thus, our method allowed for diagnosing a baseline imbalance, with or without selection for covariates, and highlights the need for adjusted statistical methods. We showed in a previous work a huge difference in the intervention effect estimate obtained from a crude analysis (without adjustment) and that obtained with multivariable regression or PS adjustment [27], and therefore confirmed that baseline imbalance occured on counfounding factors.

Moreover, the results showed that covariates significantly associated with the intervention allocation in the PS model were not the same covariates that appeared significantly unbalanced with univariate tests. Indeed, polyarthritis and radiological grade were significant in the PS model at a 5 % significance level, whereas the WOMAC score was no longer significant. These results were explained by the correlation patterns between covariates, which suggests that global approaches for the diagnosis of baseline imbalance may add some global information on relationships among covariates, missing with the univariate approach.

### Example 2: Standardized consultation for patients with OA of the knee

The PS model was built from the 26 covariates described in Table 3. The PS distribution in the two arms was not layered (see Additional file 1: Appendix C Figure 2b). However, the estimated *c-statistic* was 0.684 and the threshold value was 0.696, so the method did not detect imbalance between groups. This situation is close to the case in which the number of unbalanced covariates was small as compared to the total number of covariates, and thus, a pre-selection of covariates is needed. Therefore, we applied the selection strategy previously proposed. From Table 3, Five covariates had a standardized difference <5 *%* (physical exercise level [PEL] scale, WOMAC score, global assesment of the disease, current use of SYSADOA and use of walking sticks). Then, we estimated the correlation matrix (Pearson’s correlation coefficients were used, both for qualitative and quantitative covariates). Among the five balanced covariates, two showed correlation >0.2 in absolute value, with at least one covariate with a standardized difference >5 *%*: WOMAC score was correlated with SF-12 physical score (*r*=−0.513) and PEL was correlated with sex (*r*=0.289) and WOMAC score (*r*=−0.245). Therefore, we removed only the global assessment of the disease status, the current use of SYSADOA and the use of walking sticks from the PS model. The estimated *c-statistic* for the PS model with 23 covariates remained 0.684. However, the provided threshold value was 0.682. Consequently, after a pre-selection of covariates, a baseline imbalance was detected. This example also showed that our selection method allow for retaining a large amount of covariates, keeping the advantage of a global method over univariate testing. In the original paper, authors used an Inverse Probability of Treatment Weighted (IPTW) estimator to correct for baseline imbalance.

## Discussion

In this paper, we provide a new tool, based on the *c-statistic* of the PS model, to detect baseline imbalance in CRTs. This tool performed well for CRTs with a large sample size and a large number of covariates and allowed allowed us to capture global information, in contrast to univariate tests. In the first motivating example, our method revealed a predictor of intervention allocation that univariate methods ignored, and confirmed the presence of imbalance and the requirement of adjusted statistical methods when estimating the intervention effect. The efficiency of the proposed pre-selection strategy was shown in the second motivating example. Even if a subset of covariates was retained, the subset was still meaningful for a global approach because the pre-selection method aimed at retaining the correlation patterns between covariates.

In practice, this approach can be viewed as a kind of hypothesis testing because it relies on a “known” probability distribution and uses a threshold value defined according to a significance level (5 % in our study because we used the 95^th^ percentile of the *c-statistic* distribution). Of note, we used the 95^th^ percentile of the *c-statistic* distribution under the hypothesis of no systematic imbalance to allow us to compare the results with classical univariate tests; however, to detect smaller baseline imbalances, smaller percentiles could be adopted, especially in CRTs with a large sample size with less chance variation in baseline covariates expected. Indeed, adjustment on balanced covariates does not have a negative impact such as omitting an unbalanced risk factor would, and thus this method will be less restrictive with a smaller percentile. Moreover, as for classical tests for which a *p*-value close to 5 *%* has to be interpreted carefully, estimated *c-statistics* close to their threshold values do not necessarily mean that there is no confounding bias (if *c*<threshold) or a systematic bias (if *c*>threshold). In these situations, a risk of bias can be suspected and further considerations about the link between covariates and outcome are needed to assess the risk of bias. But again, unnecessary adjustment would have a smaller impact on the analysis that the omission of a confounder in the analysis. Statistical testing is not recommended in individually randomized trials because they are not theoretically prone to confounding bias [11]. However, as previously explained, this assumption does not hold in CRTs that randomize clusters before selecting participants. Therefore, the quantitative approach proposed in this paper could be useful to improve both the reporting of baseline characteristics and the subsequent statistical analysis.

The performance of our method was high for *n*=500, a sample size close to that observed in practice (the interquartile range of sample size per arm being [143–866]) in a recent systematic review [42]. For *n*=100, i.e. a value below the first quartile of the observed sample size per arm, the performance was low or moderate. In these situations that are highly prone to chance imbalance, covariate adjustment may be useful even if our method does not lead to the conclusion of a baseline imbalance. Our method must be viewed first as a tool to assess the risk of confounding bias and then to help identify CRTs in which an adjustment is needed, but for small sample sizes, covariate adjustment should be systematic, considering the high risk of sample fluctuations.

A limitation of this tool is the focus on ‘overt bias’ only, i.e. it can only assess the imbalance on observed characteristics as defined by Rosenbaum [49]. However, most trials collect information on a large number of baseline covariates, and given the fact that there are likely to be associations between different covariates, it is unlikely for the observed baseline covariates to be balanced between treatment arms, but for the unobserved covariates to be imbalanced. This would only happen if the observed and unobserved covariates were independent from each other and the association of these variables with the outcome variable is weak. Moreover, this tool can only help in assessing confounding bias, but not selection bias (i.e. difference in characteristics between recruited and not recruited patients). In order to detect selection bias, baseline characteristics of patients not recruited would be necessary, such as screen log data, but these data are often not available.

Further research is needed to assess the performance of the proposed method in a wider variety of situations. This study focused mainly on individual baseline characteristics: because clusters are the randomization unit, systematic imbalance on cluster-level covariates should not occur, provided the randomization method has been implemented correctly with appropriate allocation concealment, but chance imbalance on these covariates may occur. In particular, chance imbalance is likely to occur with only few randomized clusters, which is frequent; a systematic review showed that the median number of randomized clusters is 34 [9].

## Conclusion

To avoid a risk of confounding bias, CRTs should, if possible, be designed to respect the usual chronology of randomized trials (recruitment and then randomization of clusters). However it is not always feasible in practice, for example when participants are incident cases. When clusters are randomized prior to participants being recruited, the proposed method is a helpful qualitative tool to assess the risk of bias in CRTs and to provide guidance for covariate adjustment.

## Additional file

Additional file 1
**Appendix.** Simulation plan and distribution of the *c-statistic* without baseline imbalance. The R code to compute threshold values is available on request to the corresponding author. (PDF 279 Kb)
