# Supplementary material for: Propensity score to detect baseline imbalance in cluster randomized trials: the role of the c-statistic
Source: BMC Med Res Methodol. 2016 Jan 22;16:9. doi: 10.1186/s12874-015-0100-4 (PMC4724161; doi:10.1186/s12874-015-0100-4)
Supplement: Additional file 1 — Appendix. Simulation plan and distribution of the c-statistic without baseline imbalance. The R code to compute threshold values is available on request to the corresponding author. (PDF 279 Kb) [file 12874_2015_100_MOESM1_ESM.pdf]

RESEARCH

Propensity score to detect baseline imbalance in cluster randomized trials: the role of the c-statistic - APPENDIX

Clémence Leyrat[1,2,3*], Agnès Caille[1,2,3,4], Yohann Foucher[5] and Bruno Giraudeau[1,2,3,4]

*Correspondence:
clemence.leyrat@univ-tours.fr
[1]INSERM U1153, Paris, France
Full list of author information is available at the end of the article

APPENDIX A:
Distribution of the *c-statistic* without baseline imbalance and estimation of the threshold value

Objectives

The objectives were to study the distribution of the *c-statistic* in CRTs without baseline imbalance in a variety of situations to determine whether a specific threshold depending on sample size and number of covariates for each scenario is needed, rather than a unique threshold value.

Methods

Data generation

Data were generated in the same way than in the simulation study presented in the main paper, except that the r generated covariates were balanced.

Studied scenarios

We studied 36 scenarios in which the following parameters were varied:

- **the sample size per arm**, $n = (100, 500)$. In CRTs, the median number of subjects per arm is 329 (IQR=[143-866]) [1]. Thus, the chosen values correspond to the situation of a small and average size CRT.
- **the number of clusters per arm**: $k = (5, 10, 50)$,
- **the number of covariates**: $r = (4, 10, 20)$ for $n = 100$ and $r = (10, 20, 50)$ for $n = 500$, corresponding to a ratio $\frac{n}{r} = (25, 10, 5)$ for $n = 100$ and $\frac{n}{r} = (50, 25, 10)$ for $n = 500$. We considered $r_c = r_b = \frac{r}{2}$.
- **the trial design**: individually randomized trial (without clustering for the covariates $\boldsymbol{X_0}$) or CRT (with an ICC for the covariates $\boldsymbol{X}$).

Results

The boxplots below show the distribution of the *c-statistic* without systematic imbalance. Results were pooled over the number of clusters per arm k and type of trial (CRT *vs.* individually randomized trial) because these parameters had no impact on the average *c-statistic* (data not shown). Moreover, the correlation between

covariates had no impact on the *c-statistic* distribution (data not shown) when the covariates were balanced. Indeed, the correlation among covariates usually affects the *c-statistic* only when these covariates are predictors in the model.

The results showed that even in absence of systematic baseline imbalance, the median *c-statistic* varied substantially across the studied scenarios. A predictive model with a *c-statistic* > 0.7 is commonly considered to be discriminant [2]. This value increased with the number of covariates because small chance imbalance on each covariate may lead to increased global imbalance. Conversely, the median *c-statistic* decreased when the sample size increased: randomization ensures group comparability according to the law of large numbers. Chance imbalance can occur especially for small sample size [3] and then may artificially increase the *c-statistic*. Therefore, these results confirm the need for a specific threshold value for a given CRT rather a unique threshold value.

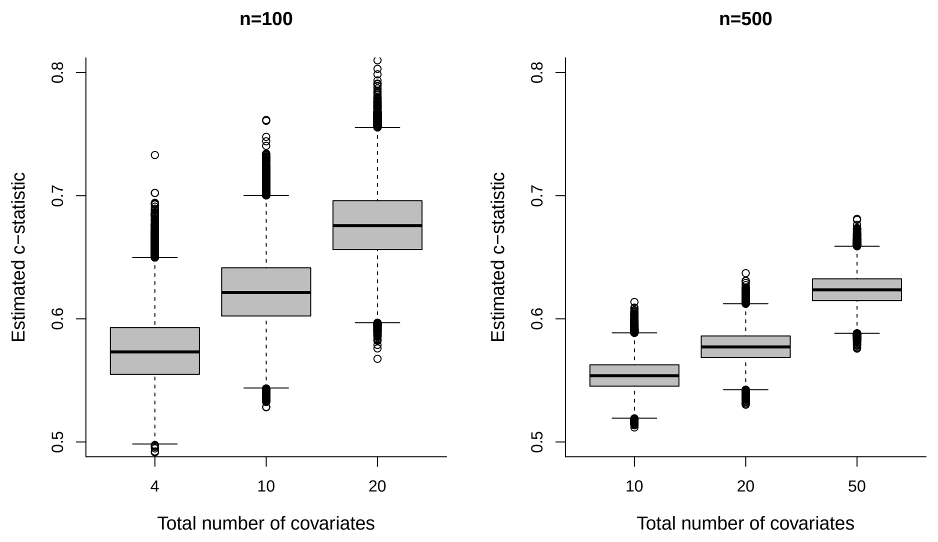

Figure 1 Distribution of the *c-statistic* of the propensity score model under the hypothesis of no baseline imbalance as a function of covariates r and the sample size (n is the sample size per arm). The type of trial (cluster randomized trial [CRT] *vs.* individually randomized trial) and the number of clusters per arm k had no effect on the distribution. 100 000 simulations were performed per scenario.

APPENDIX B: Supplementary example: internal validity

We applied our method on a third example in which there is no risk of confounding bias because of the recruitment of participants prior to the randomization of clusters, in order to control the internal validity of our method.

Oral ivermectin for difficult-to-treat head lice
This study was a double-blind double-dummy CRT comparing oral ivermectin and malathion lotion for difficult-to-treat head lice [4]. In all, 376 households were randomized, corresponding to 398 patients who received ivermectin and 441 who received malathion. Seven individual characteristics were observed at baseline (table 1). Here, the usual chronology of a randomized trial was observed: once an eligible patient was identified and recruited as an index patient, households, rather than the patients, were randomized. Thus, this CRT had low risk of confounding bias, which was strengthened by nonsignificant univariate test results and small standardized differences.

Results

The PS model contained the seven covariates displayed in Table 1 of this appendix. The PS distribution per arm is displayed in Figure c in Appendix C. Without covariate selection, our method provided a threshold value of 0.584, whereas the estimated *c-statistic* for this dataset was 0.576. With a pre-selection of covariates, five covariates among seven were retained, thus leading to an estimated *c-statistic* of 0.572 and a threshold value of 0.575. Thus, our method showed good specificity here, with or without covariate selection, because it did not lead to the diagnosis of any baseline imbalance due to recruitment or chance in a large CRT with very low risk of confounding bias.

Table 1 Patient baseline characteristics of the study comparing oral ivermectin and malathion lotion in difficult-to-treat head lice.

**Characteristics**		**Ivermectin** $n_0 = 398$	**Malathion** $n_1 = 414$	**p**	**SDiff (%)**
		mean (standard deviation)	*mean (standard deviation)*		
**Age (years)**		14.0 (11.6)	14.2 (12.0)	0.8408	1.36
**Weight (kg)**		40.4 (21.7)	40.3 (19.9)	0.9454	0.48
		n(%)	*n(%)*		
**Sex (Male)**		53 (13.3)	53 (12.8)	0.8277	1.54
**Race**	Asian	69 (17.3)	48 (11.6)	0.0594	16.40
	Black	1 (0.3)	0 (0.0)		7.08
	White	323 (81.2)	361 (87.2)		16.61
	Other	5 (1.3)	5 (1.2)		0.18
**Hair density**	Thin	59 (14.8)	50 (12.1)	0.1762	8.04
	Average	164 (41.2)	156 (37.7)		7.23
	Thick	175 (44.0)	208 (50.2)		12.59
**Hair length**	Above earlobe	65 (16.3)	57 (13.8)	0.5088	7.16
	Between earlobe and shoulder	70 (17.6)	69 (16.7)		2.44
	Below shoulder	263 (66.1)	288 (69.6)		7.48
**Live lice (< 12)**		265 (66.6)	268 (64.7)	0.5792	3.90

SDiff: standardized difference. p: p-value for univariate tests (t test for quantitative variables, adjusted chi-square test for qualitative variables to take the clustering into account).

APPENDIX C: Distribution of PS distribution per group in the 3 examples

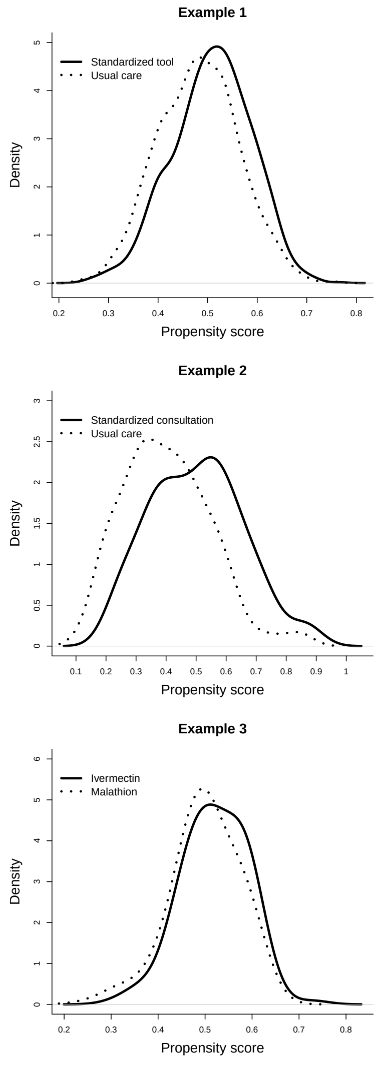

Figure 2 Propensity score distribution per arm for the three examples. **a** Example 1: standardized evaluation tool (n=1 462) *vs.* usual care (n=1 495) for the management of osteoarthritis. **b** Example 2: standardized consultation (n=154) *vs.* usual care (n=182) for patients with osteoarthritis of the knee. **c** Example 3: ivermectine (n=398) *vs.* malathion (n=441) for difficult-to-treat head-lice.

Author details
[1]INSERM U1153, Paris, France. [2]INSERM CIC 1415, Tours, France. [3]CHRU de Tours, Tours, France. [4]Université François-Rabelais, PRES Centre-Val de Loire Université, Tours, France. [5]SPHERE (EA 4275): Biostatistics, Clinical Research and Subjective Measures in Health Sciences, Université de Nantes, Nantes, France.

References

1. Ivers NM, Taljaard M, Dixon S, Bennett C, McRae A, Taleban J, et al. Impact of CONSORT extension for cluster randomised trials on quality of reporting and study methodology: review of random sample of 300 trials, 2000-8. BMJ. 2011;343:d5886–d5886.
2. Swets JA. Measuring the accuracy of diagnostic systems. Science. 1988;240(4857):1285–1293.
3. Chu R, Walter SD, Guyatt G, Devereaux PJ, Walsh M, Thorlund K, et al. Assessment and Implication of Prognostic Imbalance in Randomized Controlled Trials with a Binary Outcome – A Simulation Study. PLoS ONE. 2012 May;7(5):e36677.
4. Chosidow O, Giraudeau B, Cottrell J, Izri A, Hofmann R, Mann SG, et al. Oral ivermectin versus malathion lotion for difficult-to-treat head lice. The New England Journal of Medicine. 2010 Mar;362(10):896–905.
